# *Leptin receptor* polymorphism Gln223Arg (rs1137101) in oral squamous cell carcinoma and potentially malignant oral lesions

**DOI:** 10.1186/2193-1801-3-683

**Published:** 2014-11-22

**Authors:** Patrícia Luciana Batista Domingos, Lucyana Conceição Farias, Camila Santos Pereira, Geórgia das Graças Pena, Tatiana Carvalho Reis, Rosângela Ramos Veloso Silva, Carlos Alberto de Carvalho Fraga, Marcela Gonçalves de Souza, Mariana Batista Soares, Kimberly Marie Jones, Elytania Veiga Menezes, Sérgio Avelino Mota Nobre, João Felício Rodrigues Neto, Alfredo Maurício Batista de Paula, Jorge Gustavo Velásquez-Meléndez, André Luiz Sena Guimarães

**Affiliations:** Department of Dentistry, Universidade Estadual de Montes Claros, Montes Claros, Minas Gerais Brazil; Department of Biology, Universidade Estadual de Montes Claros, Montes Claros, Minas Gerais Brazil; Dean of Research, Associação Educativa do Brasil (SOEBRAS), Montes Claros, Minas Gerais Brazil; Department of Medicine, Universidade Estadual de Montes Claros, Montes Claros, Minas Gerais Brazil; School of Nursing, Universidade Federal de Minas Gerais, Belo Horizonte, MG Brazil

**Keywords:** Leptin, Polymorphism, SNP, *Leptin receptor*, Oral squamous cell carcinoma, Potentially malignant oral lesion

## Abstract

The purpose of this study was to assess the *LEPR* gene Gln223Arg polymorphism (rs1137101) in oral squamous cell carcinoma (OSCC) and in potentially malignant oral lesions (PMOL) in comparison to normal oral mucosa in a Brazilian population. Smokers (n = 89) were selected from a representative sample of 471 individuals from the general population of Montes Claros, Brazil. Participants were age and gender matched to patients with OSCC (n = 25) and oral epithelial dysplasia (n = 25). We investigated the *LEPR* Gln223Arg polymorphism (A>G; rs1137101) in these groups. Genotype variants were assessed by RFLP-PCR, using MspI (HPAII) restriction endonuclease. The institutional review board of the Universidade Estadual de Montes Claros approved the study (process number 2667/2011). Written informed consent for this study was obtained from all participants. The GG genotype (Arg223Arg) appears to be the more relevant polymorphic variant in OSCC. It occurred, approximately, twice as frequently in OSCC patients than in the general population. In contrast, the A allele in its homozygosis form (Gln223Gln) is significantly associated with the development of PMOL; 80% of the samples from the PMOL group exhibit AA genotype. Our findings suggest new insights regarding *LEPR* gene variations in the development of OSCC and PMOL.

## Introduction

Leptin (LEP) is a protein of the cytokine family, comprised of 167 amino acids. It is mainly synthesized in adipose tissue and is related to energy metabolism and body weight through hypothalamic regulation. Its function is achieved when it binds to its receptor, the *Leptin receptor* protein (LEPR) (Ahima and Osei 2004). In addition, the *LEP* gene has been linked to the other physiologic and pathologic mechanisms, such as bone remodeling, inflammation, rheumatoid arthritis and the development of some cancers (Ribeiro et al. 2004; Okobia et al. 2008; Beccari et al. 2013). The activated signaling pathway and proteins formed by LEP-LEPR linking has been associated with cancer pathogenesis due to interactions with Janus kinase tyrosine (JAK-2), suppressors of cytokine signaling (SOCS), and the signal transducer and activator of transcription-3 (STAT) (Fruhbeck et al. 1998; Ahima and Osei 2004).

The identification of *Leptin receptor* antagonists capable of blocking the effects of Leptin could lead to the development of new therapeutic procedures for the treatment of various types of cancer. One recently developed peptide was associated with the inhibition of several Leptin-induced pathway in breast and colorectal cancer, including JAK/STAT3, MAPK/ERK1/2 and PI3K/AKT, cyclin D1, and E-cadherin (Beccari et al. 2013). Furthermore, polymorphisms in the *LEP* and *LEPR* genes have been associated with several cancer types, such as colon, prostate, and breast cancers (Ribeiro et al. 2004; Okobia et al. 2008; Slattery et al. 2008). In prostate cancer, analysis of *LEP* polymorphism rs7799039 demonstrated that the A allele was associated with higher expression of the *Leptin receptor* (Ribeiro et al. 2004). Leptin has also been reported to interfere with the expression of oncogenic c-myc and anti-apoptotic bcl-2, regulate cell turnover and facilitate the progression of cervical cancer (Yuan et al. 2013).

The *LEPR* polymorphism Gln223Arg is characterized by an A to G transition in codon 223, resulting in a glutamine to arginine substitution (Chung et al. 1997). Higher levels of Leptin binding activity were shown in homozygous individuals for the G allele (Arg223Arg) (Quinton et al. 2001). However, there were contrary results of lower Leptin binding to the G allele in a study involving postmenopausal White women (Stefan et al. 2002).

The *LEPR* 223Arg allele has been associated with increased risk of breast cancer (Okobia et al. 2008). Similarly, in hepatocellular carcinoma (HCC), the 223Arg/Arg genotype had a significantly higher risk for HCC than the 223Gln/Gln genotype (Li et al. 2012).

Few studies have been performed to explain the significance of *LEP* and *LEPR* genes in oral malignances (Yapijakis et al. 2009; Gharote and Mody 2010). Therefore, in order to obtain better knowledge about the *Leptin receptor* gene in oral cancer, we evaluated polymorphic variants of *LEPR* Gln223Arg in the mucosa of patients with OSCC and PMOL compared to normal oral mucosa.

## Patients and methods

### Study and design

We performed a case-control study to verify a possible association between polymorphisms of the *Leptin receptor* gene (GenBank: U59251 - Nucleotide Database of National Center for Biotechnology Information, U.S.A) and OSCC and/or PMOL. To test our hypothesis, we first performed a study with a sample of non-institutionalized adults from the city of Montes Claros, Minas Gerais, Brazil. Montes Claros has approximately 361.915 inhabitants and 95.1% of them live in the urban area. Sample size calculation was performed to estimate the number of participants need for a representative sample of the municipal population (confidence level 95%, standard error <4% and event prevalence 50.0%). From a representative sample of 724 individuals, we selected only smoking individuals without OSCC or PMOL (n = 89), who were matched for age, sex, and smoking habits with OSCC (n = 25) and oral epithelial dysplasia patients (n = 25). Diagnosis of OSCC and PMOL in participants was confirmed through histopathologic analysis. In this study, only oral epithelial dysplasia was considered as PMOL. The study was approved by the institutional review board of the Universidade Estadual de Montes Claros (process number 2667/2011). Written informed consent for this study was obtained from all participants.

### *Leptin receptor*genotyping

DNA was extracted from oral mucosa scrapings from a control group and from the lesions of participants diagnosed with OSCC or PMOL. DNA samples were isolated using silica particles, which absorb the DNA. Subsequently, DNA was washed to remove impurities and was eluted in TE buffer, as previously described (Guimaraes et al. 2007). DNA from paraffin embedded tissue was extracted as previously described (Fraga et al. 2012; de Carvalho Fraga et al. 2013).

**Figure 1:**
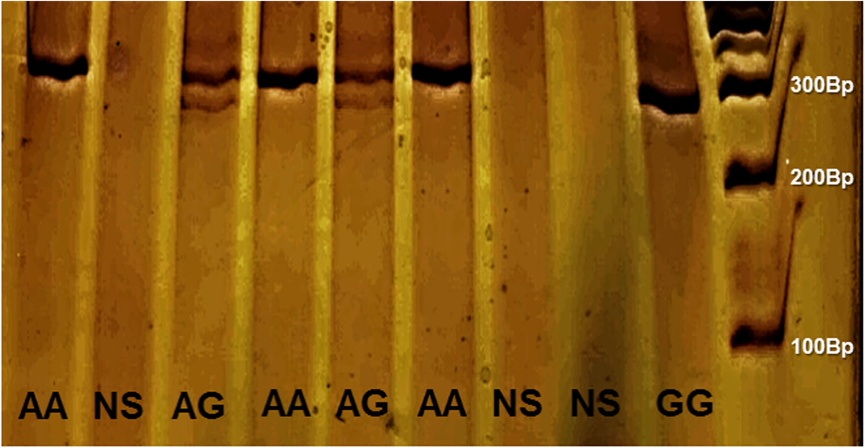
**RFLP gel electrophoresis gel.** “NS” means no sample was added.

*Leptin receptor* gene polymorphism Gln223Arg (A>G; rs1137101) was assessed by RFLP-PCR. Polymerase chain reaction for the *LEPR* gene was performed using 500 ng of genomic DNA as a template. Other reagents were used, including 4 μM of each of the following primers: F: 5′-ACCCTTTAAGCTGGGTGTCCCAAATAG-3′; R: 5′CAATATTTATGGGCTGAACTGACATT-3′; 330 bp), 0.1 mM of each DNTP (Amersham Biosciences, Pittsburgh, PA, USA), 1X PCR buffer, 2.5 mM magnesium chloride, and 2.5 U of Platinum Taq DNA polymerase (Invitrogen Life Technologies, Carlsbad, CA, USA). The 330 bp PCR product was digested with MspI (HPAII) restriction endonuclease (Fermentas Life Sciences, Lithuania) that recognizes the restriction site (C/CGG). For this SNP, the A allele lacks a MspI restriction site. Thus, individuals carrying the A allele show only one PCR band (330-bp), while carriers of the G allele show two bands (293 and 37 bp) (Figure 1). 10 μl of amplified DNA was digested with 1.0 U of MspI for 16 h at 37°C. PCR and restriction reactions were performed in a thermocycler (Eppendorf AG, Hamburg, Germany). The PCR products were visualized by electrophoresis in 10% acrylamide gel stained with silver. Positive control for digestion reaction was used.

### Statistical analysis

The Chi-square test was used to compare genotypic distributions of *Leptin receptor* gene polymorphism Gln223Arg and clinical data in the studied groups. Statistical analyses were carried out using SPSS 17.0 software. Probability values <0.05 were considered statistically significant.

## Results

Clinical data and group distributions are displayed in Table 1. Histologic gradation of OSCC and PMOL was not considered in this study. Lesions were only histologically classified as OSCC and PMOL. Table 2 shows the genotype distribution of the *LEPR* polymorphism by group. The A allele in its homozygosis state (Gln223Gln) shows significant association with PMOL; 80% of the samples exhibit the AA genotype. On the other hand, the GG genotype (Arg223Arg) appears to be the more common polymorphic variant in OSCC. It occurred, approximately, twice as frequently in OSCC patients than in the control group. The control group had higher frequencies of AG and AA variants. The GG genotype showed lower distribution in these samples; the A allele seems to be more important in this group. No association between gender and *LEPR* genotype variants were observed (Table 3). No associations were observed among controls, PMOL, OSCC and clinicopathological variables.

**Table 1:** **Distribution of samples by age and gender**

Clinical parameters	Sample groups		
	General population/smokers	PMOL	OSCC
	(n = 89)	(n = 25)	(n = 25)
Gender			
Male	67 (75.3%)	17 (68.0%)	21 (84.0%)
Female	22 (24.7%)	08 (32.0%)	04 (16.0%)
Age (in years)			
Variation	28-92	36-82	28-77
Mean ± SD	55.9 ± 13.9	58.1 ± 12.0	58.0 ± 13.6

**Table 2:** **Frequency of** ***LEPR*** **genotypes in normal oral mucosa, potentially malignant oral lesions, and oral squamous cell carcinoma**

	LEPR genotype		
Group	GG (%)	AG (%)	AA (%)
General population/smokers	11 (12.4)	38 (42.7)	40 (44.9)
Potentially malignant oral lesion	01 (4.0)	04 (16.0)	20 (80.0)
OSCC	06 (24.0)	07 (28.0)	12 (48.0)
*p*-value		0.011*	

*Analyzed by X^2^ test. Significant *p*-value < 0.05.

**Table 3:** ***LEPR*** **genotypes associated with gender in normal oral mucosa, potentially malignant lesions, and oral squamous cell carcinoma**

	General population/smokers			Potentially malignant lesion			OSCC		
Variables	GG (%)	AG (%)	AA (%)	GG (%)	AG (%)	AA (%)	GG (%)	AG (%)	AA (%)
**Gender**									
Male	07 (63.6)	33 (86.8)	27 (67.5)	01 (100.0)	03 (75.0)	13 (65.0)	05 (83.3)	07 (100.0)	09 (75.0)
Female	04 (36.4)	05 (13.2)	13 (32.5)	0 (0.0)	01 (25.0)	07 (35.0)	01 (16.7)	0 (0.0)	03 (25.0)
*p*-value*		0.089			0.725			0.357	

*Analyzed by X^2^ test. Significant *p*-value < 0.05.

## Discussion

Leptin (LEP) is a hormone secreted from adipocytes that plays an important role in energetic metabolism, control of food intake, and obesity through its binding to LEPR receptors (Dutta et al. 2012). Leptin receptors are mainly found in the hypothalamus. However, some studies indicate that other tissues may produce this protein, such as skeletal muscle, liver, kidney, bone marrow, lung (Margetic et al. 2002), and placenta (Zhao et al. 2003). An isoform of the *LEPR* gene was also expressed in oral keratinocytes (Groschl et al. 2005). It has been suggested that there is an association between *Leptin receptor* Gln223Arg polymorphisms and increased risk for oral cancer (Yapijakis et al. 2009; Gharote and Mody 2010). Related studies from out laboratory have found associations between metabolic genetic markers and OSCC (Correa et al. 2011; Fonseca-Silva et al. 2012; Fraga et al. 2012), a fact that prompted us to investigate whether there is a difference between frequencies of variants of the Gln223Arg polymorphism between case and control groups.

The *LEPR* Gln223Arg polymorphism, resulting in a change of glutamine to arginine at codon 223 in exon 6 of *Receptor Leptin* gene, has been associated with the development of and increased risk for cancer. In breast cancer, for example, *LEPR* 223Arg was associated with increased serum Leptin levels (Quinton et al. 2001). However, studies are in conflict regarding which variant of the *LEPR* Gln223Arg polymorphism is associated with a higher risk of cancer. A meta-analysis study suggested that the presence of the A allele (*LEPR* Gln223) is a risk factor in breast cancer (Wang et al. 2012). However, other similar research indicated that a higher frequency of the G allele (*LEPR* 223Arg) was associated with increased risk. Additionally, the risk of breast cancer associated to the G allele differed based on ethnicity (Liu and Liu 2011).

In this study, we hypothesized that variants of the *LEPR* Gln223Arg polymorphism can be associated with oral squamous cell carcinoma and PMOL. Despite the small group of cases, our results were able to demonstrate a significantly increased frequency of the GG genotype (Arg223Arg) in cancer patients in comparison to the control group. The GG genotype was twice as common in the OSCC and PMOL group than in the control group. A previous study showed that breast cancer patients with the G allele were more likely to develop poorly differentiated tumors than those with the A allele (Gu et al. 2012). However, it has been suggested that the A allele (*LEPR* Gln223) is an important risk factor in breast cancer (Wang et al. 2012). The GG genotype of the *LEPR* Gln223Arg polymorphism was significantly prevalent in non-small cell lung cancer (Li et al. 2012). We found only one published study that investigated the *LEPR* Gln223Arg polymorphism in oral cancer (Yapijakis et al. 2009). Similar to our findings, these investigators also found a higher frequency of the GG genotype in subjects with oral cancer.

PMOL is a lesion with potential for becoming squamous cell carcinoma (Napier and Speight 2008). Studies have focused on the investigation of molecular, genetic, and epigenetic alterations in these oral lesions, such as *p16* methylation (Fonseca-Silva et al. 2012), genetic polymorphisms (Fraga et al. 2012), microRNAs (Brito et al. 2013) and protein expression restricted to malignant cells as MAGE-A (Ries et al. 2012). Analysis of *LEP-LEPR* in PMOL was not previously investigated. Therefore, we examined frequencies of the *LEPR* Gln223Arg polymorphism in the mucosa of OSCC and PMOL patients and a control group without the disease. The A allele in its homozygosis state (Gln223Gln) was significantly associated with PMOL with 80% of the samples exhibiting the AA genotype. This is the first report investigating the relationship between *LEPR* polymorphism and OSCC and PMOL. Other studies are needed to understand the role of *LEPR* variants in the development and progression of oral cancers, especially in relation to Leptin levels or in association with the stages of dysplasia. It is important to highlight that considering the complexity of gene expression control, it is not possible to know whether the results are influenced by other environmental variables involved.

In conclusion, our findings suggest a need for further investigation of the relationship between variants of the *LEPR* gene and the development and progression of oral cancer. *LEP-LEPR* signalling may be implicated in pathogenesis of these lesions. Additional studies may lead to a clearer understanding of the mechanism by which allelic variation in the Gln223Arg gene affects risk for the development and progression of oral cancer.

## Authors’ informations

Dr. Guimarães, Dr. Velásquez-Meléndez and Dr. De Paula are research fellows at the Conselho Nacional de Desenvolvimento Científico e Tecnológico (CNPq/Brazil).
